# Fetal Lower Urinary Tract Obstruction (LUTO): a practical review for providers

**DOI:** 10.1186/s40748-015-0026-1

**Published:** 2015-11-18

**Authors:** Sina Haeri

**Affiliations:** St. David’s Women’s Center of Texas, Austin Maternal-Fetal Medicine, 12200 Renfert Way, G-3, Austin, Austin, TX 78758 USA

## Abstract

Fetal lower urinary tract obstruction (LUTO) is a serious condition, which commonly results in marked perinatal morbidity and mortality. The characteristic prenatal presentation of LUTO includes an enlarged bladder with bilateral obstructive uropathy. While mild forms of the disease result in minimal clinical sequelae, the more severe forms commonly lead to oligohydramnios, dysplastic changes in the fetal kidneys, and ultimately result in secondary pulmonary hypoplasia. The aim of this review is to provide practitioners with a practical and concise overview of the presentation, evaluation, and treatment of LUTO.

## Introduction

**Fig. 1:**
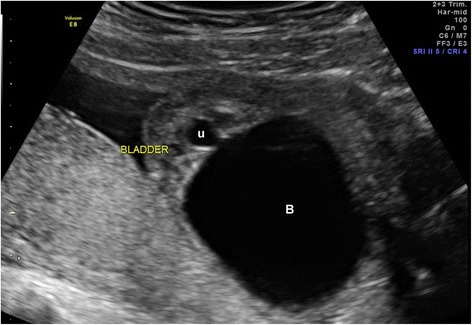
Ultrasound image of fetal bladder obstruction with the characteristic “keyhole” sign (*B: Bladder, u: urethra*)

**Fig. 2:**
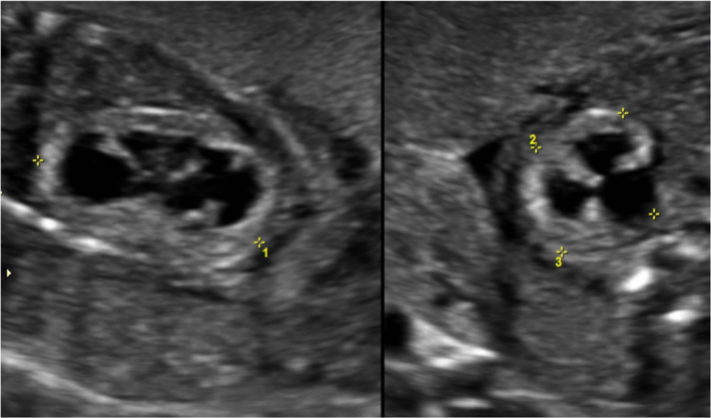
Ultrasound image of hyperechoic and small for gestational age fetal kidneys, secondary to fetal lower urinary tract obstruction

Lower urinary tract obstruction (LUTO) in the fetus can result in increased perinatal morbidity by causing abnormal development of the urinary tract along with under-development of the lungs [1, 2]. The characteristic prenatal presentation of LUTO includes an enlarged bladder (Fig. 1) with bilateral hydroureteronephrosis [1, 2]. While mild forms of the disease, as seen in functional defects, may lead to minimal clinical sequelae, the more severe forms commonly lead to oligohydramnios, a distended urinary tract, renal dysplasia (Fig. 2), as well as pulmonary hypoplasia [1, 2]. The aim of this review is to provide the practitioner with a practical and concise overview of the presentation, evaluation, and treatment of LUTO.

### Review

#### Incidence

The incidence of LUTO has been reported to be between 1 in 5,000 to 1 in 25,000 pregnancies, which may be an underestimation given that there is no accounting for cases of elective termination, intrauterine fetal demise (IUFD), or postnatal diagnosis [3, 4]. The two most common causes of LUTO include posterior urethral valves (PUVs), and urethral atresia [5]. In general, obstruction at the bladder outlet in males is caused by PUVs, whereas in females it is secondary to urethral atresia [4].

#### Presentation

The sonographic features of LUTO include marked distention of the bladder, often with a thickened wall (greater than 2 mm) [4]. A “keyhole” sign may be seen in cases of PUV, which reflects dilation of the posterior urethra, proximal to the level of the obstruction; however the “keyhole” sign (Fig. 1) is not a specific ultrasound sign of PUV and may also be present in different causes of LUTO [6]. While ureterectasis (dilation of the ureter) and caliectasis (dilation of renal calices) are common findings in cases of LUTO, it must be noted they are only present in 40–50 % of cases and their absence should not rule out the diagnosis of bladder obstruction [6]. The vesicourethral reflux from the increased intravesical pressure may lead to pan dilation of the urinary tract, and the increased pressure in turn may result in dysplastic renal changes (Fig. 2) [5]. Accordingly, the presence of subcortical cysts, small and hyperechoic kidneys, as well as absence of caliectasis should raise suspicion for end-stage obstructive uropathy [7, 8]. Long-standing oligohydramnios resulting from LUTO may lead to fetal anatomical deformities including clubfeet and Potter facies [7, 8].

#### Differential diagnosis

The differential diagnosis of LUTO is highly dependent on the fetal gender [5, 8, 9]. As mentioned earlier, in the male fetus the most likely diagnosis is PUVs; however, other rare causes such as a prolapsing uretereocele from a duplicated collecting system must be kept in mind [5, 8, 9].

In the female fetus, urethral atresia is the most common cause; however, persistent cloaca, caudal regression, and megacystis-microcolon-intestinal hypoperistalsis syndrome must be ruled out. [5, 8, 9]. A persistent cloaca generally presents with an enlarged bladder prior to 16 weeks gestation, and will often accompany presence of debris within the cloaca, and intraluminal calcifications within bowel loops (due to intestinal communication) [2, 10–13]. Caudal regression generally presents with normal amniotic fluid volume, vertebral and lower extremity defects, and bladder extrophy. Megacystis-microcolon-intestinal hypoperistalsis syndrome again presents with normal to high amniotic fluid volume, dilated bladder with a thin wall, as well as dilated loops of bowel [13].

#### Evaluation

Initial evaluation of the fetus with suspected LUTO should include a comprehensive anatomic survey and echocardiogram to rule out any co-existing abnormalities, gender determination, amniotic fluid volume assessment, as well as diagnostic genetic evaluation given that over 10 % of LUTO cases are associated with Trisomies 13, 18, or 21 [2, 5, 12, 13]. Given the latter point, it is highly recommended that the parents meet with a genetic counselor as part of the diagnostic and treatment process.

In non-isolated cases of LUTO, strong consideration should be given to an underlying genetic process, and invasive prenatal diagnosis initiated as the presence of a genetic abnormality may preclude candidacy for fetal intervention [14, 15].

In isolated cases of LUTO with normal amniotic fluid volume, an amniocentesis should be offered to rule out an underlying genetic disorder [2, 13]. Otherwise, evaluation should consist of serial assessments of the fetal anatomy (every 1–2 weeks) to rule out late developing abnormalities (e.g. oligohydramnios, renal dysplasia) [16]. As discussed later, normal amniotic fluid volume generally denotes a milder form of disease, and does not require fetal intervention [2, 16].

In isolated LUTO cases with oligohydramnios, a thorough discussion should be held with the parents before proceeding with invasive evaluations of the fetus, to ascertain the parental wishes for intervention [9, 12]. Should the parents elect to terminate the pregnancy, genetic testing should be highly recommended as in some cases (e.g. Megacystis-microcolon-intestinal hypoperistalsis syndrome) the recurrence risk might be as high as 25 % (due to autosomal recessive inheritance) [5, 17–20]. Should the parents elect intervention, invasive testing (as outlined below) should be undertaken to assess renal function, and genetic make up of the fetus.

Evaluation of the fetus should begin with two consecutive diagnostic vesicocenteses (bladder taps) [21–24]. Under ultrasound (US) guidance, the bladder should be visualized in its largest dimensions, and a point of entry (preferably avoiding the placenta) should be identified, where the needle tip is ideally placed in the lower aspect of the bladder [23, 24]. Care should be taken (e.g. use of color Doppler) to avoid injury to intra-abdominal structures such as the umbilical arteries. Using a 22 gauge spinal needle, the bladder should be drained as much as safely and technically possible. This first specimen should be sent for genetic evaluation including a complete genomic hybridization microarray. A recent report demonstrated a 100 % result rate from fetal urine specimens, obviating the need for an amniocentesis or placental biopsy [15]. Excess urine from the first drainage may be sent for electrolyte evaluation as well, provided that there is enough volume to complete the genetic evaluation as well. It must be cautioned that unfavorable electrolyte analysis should not be viewed as an exclusion criterion for intervention, and electrolyte analysis from a second drainage should be sent, as stagnant urine may not accurately reflect the correct renal function. The second vesicocentesis should be performed 24–48 h later in similar fashion, and the urine sample sent for sodium (Na), chloride (Cl), osmolarity (Osm), calcium (Ca), and beta-2 microglobulin (β_2_) [23, 24]. The prognostic criteria suggested by Glick & colleagues, [25–27] as outlined in Table 1, along with the sonograophic appearance of the kidneys should be used in assessing candidacy for intervention. For a fetus to be categorized as having “good” prognostic indicators, the values in Table 1 (especially Na, Cl, Osm, Ca) should be within the favorable range, and the sonographic assessment of the fetal kidneys should not demonstrate absence of cortical cysts or hyperechogenicity.

**Table 1:** Prognostic criteria using fetal urine [25]

Urinary component	Favorable
Sodium (Na)	Less than 100 mEq per liter
Chloride (Cl)	Less than 90 mEq per liter
Osmolarity (Osm)	Less than 210 mEq per liter
Calcium (Ca)	Less than 2 mmol per liter
Beta-2 microglobulin	Less than 2 mg per liter

Before proceeding with the treatment options for LUTO, it is prudent to mention one major limitation of the above prognostic indicators in that the values are not adjusted for gestational age (fetal urine becomes more hypotonic until reaching its nadir at 20–21 weeks), nor are they reflective of postnatal renal function, therefore the parents should be cautioned regarding the possibility of poor renal function at time of birth despite intervention [23].

#### Treatment

As mentioned earlier, elective termination of the pregnancy should be discussed with couples facing a child with LUTO. Those that elect to continue the pregnancy should meet with a Pediatric Nephrologist and Urologist to review the possible postnatal courses including short and long-term outcomes (e.g. dialysis, transplantation) so that realistic expectations are set.

For fetuses with a favorable prognostic indicators (Table 1) and oligohydramnios, treatment is predominantly aimed at restoration of amniotic fluid volume for prevention of pulmonary hypoplasia, and urinary decompression for attenuation of on-going renal damage [20, 24]. Treatment options for this subset of fetuses includes vesicoamniotic shunting (most commonly used), valve ablation via cystoscopy, and vesicostomy.

**Fig. 3:**
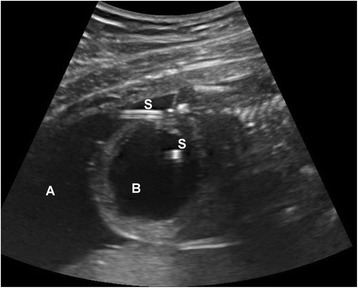
Ultrasound image of a vesicoamniotic shunt with the two catheter ends (S) in the fetal bladder (B) and amniotic cavity (A)

Vesicoamniotic shunting is a percutaneous procedure performed under ultrasound guidance, using local anesthesia for maternal pain relief [7]. Prior to placement of the shunt, an amnioinfusion (e.g. warm sterile saline infused with Nafcillin) is routinely required to allow space for deployment of the proximal end of the catheter. Fentanyl (15 μgLKg) and pancuronium (0.5–2 mg/Kg) injection into the umbilical vein or into the fetal arm muscle may be used for fetal anesthesia. A double pig tailed catheter (Rodeck/Rocket or Harrison shunts) is then placed with the distal end in the fetal bladder, and proximal end within the amniotic cavity (Fig. 3) [2]. It must be noted that due to the small caliber and long length of the shunts, complete decompression of the bladder or the urinary tract may not be seen in all cases, especially those with a high-grade obstruction. The outcomes associated with vesicoamniotic shunting are not clear [28]. Data to date have not proven reliable due to heterogenous patient populations. Furthermore, the most recent randomized trial aimed at examining the utility of vesicoamniotic shunting (PLUTO Trial) ended prematurely without answering this important question due to poor recruitment, although anecdotal evidence appears to point to improved outcomes with this intervention [5, 28].

**Fig. 4:**
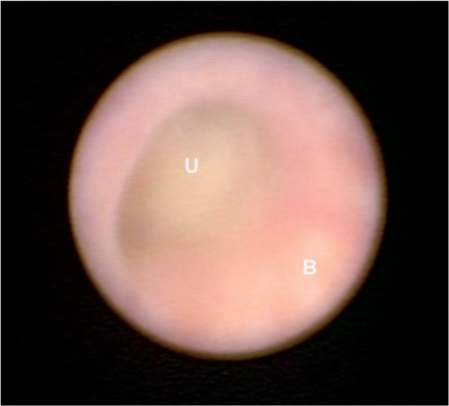
Fetal cystoscopy image within the bladder (B) demonstrating the point of obstruction at the urethra (U)

Fetal cystoscopy, which is technically more difficult than vesicoamniotic shunt placement, is an emerging treatment option for LUTO [29–31]. This option holds several advantages over shunting in that it allows for direct visualization of the obstruction to ascertain specific diagnosis (Fig. 4), and does not require an amnioinfusion [2]. Given the need for minimal maternal movement, as well as the longer procedure duration, consideration should be given for maternal regional (epidural or spinal) anesthesia, rather than local analgesia. Similar to vesicoamniotic shunting, fetal anesthesia may be accomplished by injecting fenatnyl (15 μgLKg) and pancuronium (0.5–2 mg/Kg) into the umbilical vein or into the fetal arm muscle. Using a larger trocar (2.2 mm) than used for vesicoamniotic shunting (1.6 mm), a 1.0 mm fetoscope in a curved sheath and at least a 70° field of view is used for cystoscopy [2, 5, 17, 32, 33]. After confirming that the trochar is inside the fetal bladder, the fetoscope is introduced into the sheath, and advanced toward the bladder neck and the dilated posterior urethra. If a membrane-like obstruction of the urethral lumen is seen, the diagnosis of PUV is confirmed and the valves can be treated using hydroablation, guide-wire or laser fulguration [2, 5, 17, 32, 33]. However, if a non-membrane-like structure is found, even with the fluid injection, the UA is diagnosed and no attempt to perforate this structure is performed, and a vesicoamniotic shunt is placed [2, 5, 17, 32, 33]. The main complication of fetal cystoscopic laser ablation of PUV is urological fistula, which seems to be associated with less operator experience, elevated laser power/energy and less curved instruments [34]. Therefore, percutaneous fetal cystoscopy is useful for diagnostic as well as therapeutic purposes in LUTO, however it is necessary to have adequate experience and instruments to perform this challenging procedure. Lastly, given that this procedure remains experimental, it should be performed under institutional review board approval.

**Fig. 5:**
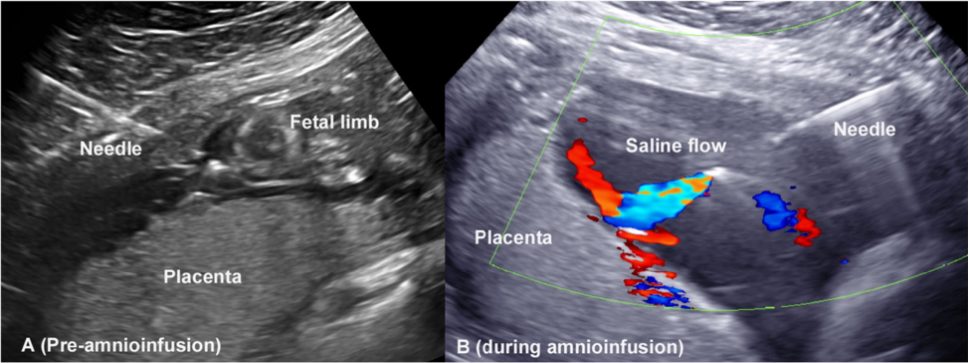
**a** pre-amnioinfusion insertion of an echo-tip 22 gauge needle into the amniotic cavity (*pocket of loops of cord adjacent to fetal limb*), and **b**) Color Doppler imaging demonstrating amnioinfusion using sterile saline

Fetal vesicostomy, via open fetal surgery, is yet another treatment option for LUTO [35, 36]. However, despite its promising neonatal results, the associated maternal and perinatal morbidity, along with the paucity of large scale data preclude it’s widespread use for the treatment of LUTO at this time. In addition, this technique does not improve the bladder function [35]. Despite the promising results for each of the above interventions, there remains a paucity of high quality data supporting the use of fetal intervention in cases of LUTO with a favorable prognostic profile and oligohydramnios.

With respect to outcomes for the abovementioned intervention, a recent review by Morris and Kilby provided a useful overview [7]. Vesicoamniotic shunting improved perinatal survival when compared with no treatment (odds ratio (OR) 3.86; 95 % confidence intervals (CI) 2.00–7.45), albeit at the expense of residual risk of poor long-term postnatal renal function (OR 0.67, 95 % CI 0.22–2.00). Similarly, cystoscopy appears to improve perinatal survival by an OR of 20.51 (95 % CI 3.87–106.89); however, when compared to shunting, there appears to be no significant improvement in perinatal survival OR 1.49 (0.13–16.97). Appropriately, they concluded that while prenatal intervention appears to improve perinatal survival, there might be a trend towards increased childhood morbidity (associated with chronically poor renal function) in the survivors, a point which should be made clear to the parents at the time of diagnosis [7].

Expectant management is yet another option for couples facing this serious problem in their child. In cases of LUTO with preserved normal amniotic fluid levels, favorable pulmonary function should be expected. The parents should meet with pediatric subspecialists to prepare for the postnatal course, which may include surgery and dialysis. In cases of LUTO with oligohydramnios, palliative care should be offered. In the event the parents decline palliative care, consultation with pediatric subspecialists, especially Neonatology should be undertaken to prepare the parents for expected complications especially pulmonary hypoplasia. Furthermore, discussions between the obstetric team and the parents should be held to review parental wishes for intervention in the event of non-reassuring fetal status considering the poor prognosis.

Lastly, consideration for intervention should be given for those fetuses with a poor prognostic profile, or end-stage fetal renal disease, which are not candidates for the above interventions [2]. While termination of the pregnancy or palliative care is the uniformly accepted recommendation for these cases, they may not be an option for some parents due to personal or religious beliefs. In such instances, under an experimental and case-by-case basis, some groups (including the author) have offered serial amnioinfusions(Fig. 5) for pulmonary palliation [37, 38]. The couple is asked to meet with Neonatology, Pediatric Urology, and Pediatric Nephrology to thoroughly review the expected outcomes (including morbidity and mortality) of a neonate with end-stage renal disease requiring dialysis and transplantation. If still interested, serial amnioinfusions are performed for oligohydramnios until 28–30 weeks, and delivery for fetal distress reserved until an estimated fetal weight of 2–2.5 kg to allow for peritoneal dialysis cathether placement candidacy. It must be noted that this intervention is experimental, and large-scale studies are needed to assess its utility and safety.

## Conclusions

LUTO can lead to marked morbidity and mortality in the fetus; therefore it is prudent for obstetric providers to understand it’s general presentation, and management principles. While several treatment modalities exist, including vesicoamniotic shunting and fetal cystoscopy, large scale studies are needed to validate their efficacy in preventing pulmonary hypoplasia, and preserving renal function.
